# Influence of source of origin and region of finishing on growth performance and carcass characteristics of finishing heifers fed in the United States

**DOI:** 10.1093/jas/skaf013

**Published:** 2025-01-23

**Authors:** Erin R DeHaan, Colten W Dornbach, Amanda D Blair, Nicole C Burdick Sanchez, Jeffery A Carroll, Paul R Broadway, Warren C Rusche, Kristin E Hales, Zachary K Smith

**Affiliations:** Department of Animal Science, South Dakota State University, Brookings, SD, USA; Department of Animal and Food Sciences, Texas Tech University, Lubbock, TX, USA; Department of Animal Science, South Dakota State University, Brookings, SD, USA; Livestock Issues Research Unit, Agricultural Research Service, U.S. Department of Agriculture, Lubbock, TX, USA; Livestock Issues Research Unit, Agricultural Research Service, U.S. Department of Agriculture, Lubbock, TX, USA; Livestock Issues Research Unit, Agricultural Research Service, U.S. Department of Agriculture, Lubbock, TX, USA; Department of Animal Science, South Dakota State University, Brookings, SD, USA; Department of Animal and Food Sciences, Texas Tech University, Lubbock, TX, USA; Department of Animal Science, South Dakota State University, Brookings, SD, USA

**Keywords:** beef, carcass, growth, Northern Plains, Southern Plains, vaginal temperature

## Abstract

The objective was to evaluate growth performance and carcass traits of finishing beef heifers sourced and finished in different regions in the U.S. Heifers (*n* = 190; initial body weight [**BW**] 483 ± 0.4 kg and 425 ± 1.9 kg for South Dakota [SD] and TX sourced, respectively) were used in a 2 × 2 factorial arrangement of origin state (SD vs. TX) and finishing state (SD vs. TX) was used. Heifers were allotted on day −1 to: 1) sourced from SD and finished in SD (SD-SD), 2) sourced from SD and finished in TX (SD-TX), 3) sourced from TX and finished in SD (TX-SD), and 4) sourced from TX and finished in TX (TX-TX). Heifers were weighed on d -1, 3, 15, 28, 56, 78 (TX-TX and SD-TX) and 90 (SD-SD and TX-SD). On day 0, SD-TX and TX-SD heifers were shipped to their respective finishing locations. The following morning (day 1), SD-TX and TX-SD heifers were individually weighed to determine transit shrink. To monitor transit stress effects, vaginal temperature probes were used on all SD-TX and TX-SD heifers and a portion of SD-SD and TX-TX heifers on day −1 and removed on day 3. Clinical attitude scores (**CAS**) were recorded on days −1, 0, 1, 2, and 3 for bovine respiratory disease symptoms. Transported heifers had decreased temperatures (*P* ≤ 0.01) during transit and post-transit and increased (*P* ≤ 0.01) vaginal temperature during loading and unloading compared to non-transported heifers. On days 0, 1, and 3 there was a shift in the distribution of heifers that had a CAS score greater than 0 for TX-TX, SD-TX, and TX-SD. Heifers endured elevated ambient temperatures (temperature-humidity index > 75) for 54% and 18% of the feeding period for TX and SD. Growth performance and carcass trait interactions were significant (*P *< 0.01) except for day −1 BW, percent shrink during transit, average daily gain, dressing percent, ribeye area, and liver abscess severity, which did not differ (*P *> 0.30). A shift in the distribution (*P *< 0.02) towards a greater proportion of Yield Grade (YG) 1 and Select carcasses was observed for TX versus SD. Overall, heifers transported to higher ambient temperatures had improved overall YGs but decreased dry matter intake, quality grades (**QG**), and limited growth recovery (45 kg lighter) following transit than non-transported heifers. Heifers transported to lower ambient temperatures recovered growth and had improved QG (*P *< 0.02) at the same thickness of rib fat compared to non-transported heifers but had decreased overall yield and yield grades.

## Teaser

Cattle performance and carcass characteristics are impacted by the source of origin, finishing region, and time of the year at which they are transported to a finishing location.

## Introduction

Beef cattle are procured from all regions of the United States, and most are finished in a feedlot for harvest. Feedlot characteristics can vary based on the region of the United States. A key characteristic that separates feedlots in the Southern Plains region from the Northern Plains region is the type of diets commonly fed. Diets fed in both regions are predominantly corn-based and commonly contain byproducts from the ethanol industry and fermented roughages and/or hay (Samuelson et al., 2016). Nonetheless, the type of corn processing between regions can vary. Cattle fed in the northern plains are commonly fed dry-rolled corn-based diets (Asem-Hiablie et al., 2016) while cattle in the southern plains are predominantly fed steam-flaked corn-based diets (Samuelson et al., 2016). Grain processing can affect how corn is digested in a beef animal. Steam-flaked corn has been reported to increase ruminal and post-ruminal starch digestibility and availability compared to dry-rolled corn (Barajas and Zinn, 1998; Corona et al., 2005). Thus, increased starch digestibility could alter subsequent growth performance and how fat is deposited in the animal.

Another difference between regions is the type of backgrounding system cattle are developed in. In the Southern Plains, the grazing period is extended because of warmer temperatures and a lower incidence of snowfall compared to the Northern Plains environment (NASEM, 2016). Thus, it is common for calves backgrounded in the Southern Plains to be placed in a forage-based setting where they receive most of their diet from grazing rather than fed in a dry lot setting with a majority of their diet coming from harvested forages. However, as concluded in a survey by Asem-Hiablie et al., (2016), 70% of operations in the Northern Plains do not graze calves in a forage-based setting but instead backgrounded calves in a dry lot setting. Diets offered in a dry lot setting include high roughage or limit-fed high-concentrate diets, which use cheaper feed energy resources on an equal energy basis (Sip and Pritchard, 1991).

Cattle originating from the Northern Plains are often shipped to other regions, such as the Southern Plains, because of the limited feedlot infrastructure available to finish cattle (Pritchard and Preston, 1992). Transportation of cattle can induce stress and lead to heightened immune responses, especially when cattle are transported for a long period of time (Arthington et al., 2003; Deters and Hansen, 2020). Transported cattle have also been reported to have slower overall growth following transit (Pritchard and Preston, 1992). Weather between feeding regions can be drastically different, especially at certain times of year. Environmental factors, such as humidity and precipitation, and whether cattle are fed in extremely low or high ambient temperatures could impact cattle performance and the competitiveness of feeding cattle between regions (Pritchard and Preston, 1992).

Information regarding the relationship of the movement of cattle between regions with vastly different production systems and differing ambient temperatures, and the degree to which these factors can influence performance is novel and requires further consideration. Therefore, it was hypothesized that transporting heifers to a finishing region different than their source of origin would negatively alter feedlot performance compared to heifers of similar origin that were not transported. To test this, this study evaluated the influence of region of finishing (SD or TX) and heifer origin (SD or TX) on growth performance, health measures, and carcass characteristics of finishing beef heifers.

## Materials and Methods

### Institutional animal care and use approval

All experimental procedures were approved by the South Dakota (SD) State University Institutional Animal Care and Use Committee (IACUC; protocol #2206-031E) and the Texas Tech University IACUC (protocol #2022-1198) and were conducted from June 2022 to October 2022.

### Cattle management

Yearling heifers (*n* = 190; initial body weight (**BW**) 483 ± 0.4 kg and 425 ± 1.9 kg for SD and TX sourced, respectively) sourced within each geographical region were used in a completely randomized design with a 2 × 2 factorial arrangement of origin state (SD vs. Texas TX]) and finishing state (SD vs. TX). Heifers (*n* = 98) in the Northern Plains region were sourced from western SD and transported approximately 513 km to the Ruminant Nutrition Center (**RNC**) in Brookings, SD. Similarly, heifers (*n* = 92) in the southern region were sourced from the TX panhandle and transported approximately 370 km to the Burnett Center in New Deal, TX. In SD, heifers were received in January 2022 (day −188) into concrete pens with bed packs and administered vaccinations against respiratory pathogens (Bovishield Gold FP5 VL5, Zoetis Inc., Parsippany, NJ), *Clostridium* species (Ultrabac 7/Somubac, Zoetis Inc.), and internal and external parasites (Dectomax, Zoetis Inc.). In March (day −130), SD heifers received a Ralgro implant (36 mg zeranol; Merck Animal Health, Madison, NJ). SD-sourced heifers were offered a limit-fed high-concentrate diet before the study initiation. On day −14, heifers in TX were received in soil-surface pens, and administered vaccinations for protection against respiratory pathogens (Myco-B One Dose, American Animal Health, Fort Worth, TX; Bovilis Vista 5Q, Merck), *Clostridium* species (One Shot Ultra 7; Zoetis Inc.), and internal and external parasites (Cydectin; Elanco, Indianapolis, IN). Texas-sourced heifers had previously grazed wheat pasture. All heifers received a Revalor-200 implant (200 mg trenbolone acetate and 20 mg estradiol; Merck Animal Health) on day 3. Heifers finished in SD were fed in concrete-surfaced pens (7.62 m × 7.62 m, with 7.62 m of bunk space) and heifers finished in TX were fed in soil-surfaced pens (4.9  × 30.5 m in length, 4.9 m of bunk space).

An allotment BW from day −14 was used to sort heifers into four treatments: 1) heifers sourced from SD and finished in SD (SD-SD, *n* = 48); 2) heifers sourced from SD and finished in TX (SD-TX, *n* = 50); 3) heifers sourced from TX and finished in SD (TX-SD, *n* = 46); 4) heifers sourced from TX and finished in TX (TX-TX, *n* = 46). Allotment procedures accounted for variations in pen size between finishing locations with 48 heifers in SD-SD and 46 heifers in TX-SD treatments (*n* = 6 pens/treatment), and 50 heifers in SD-TX and 46 heifers in TX-TX treatments (*n* = 5 pens/treatment). Transport for SD-TX and TX-SD heifers occurred on day 0. Heifers were transported approximately 1,540 kilometers simultaneously, without lairage time, for 17 h or 18 h (TX-SD and SD-TX heifers, respectively). From day 0 to study end (day 78 for heifers fed in TX and day 90 for heifers fed in SD), heifers were fed a standard grain-based finishing diet common in the respective region (Table 1 for SD finished and Table 2 for TX finished). One heifer from the SD-SD treatment was removed from the study on day 55; data from this heifer were included until the time of removal.

**Table 1.** Diet composition of heifers fed in SD for 90 d^1^

	Days 1 to 7		Days 8 to 12		Days 13 to 35		Days 36 to 90	
Item	SD-SD	TX-SD	SD-SD	TX-SD	SD-SD	TX-SD	SD-SD	TX-SD
Dry-rolled corn^2^, %	48.03	39.24	48.02	48.02	60.09	60.09	69.36	69.36
Corn silage, %	32.17	32.64	32.16	32.16	20.53	20.53	—	—
Oatlage, %	—	—	—	—	—	—	10.00	10.00
Grass hay, %	—	8.05	—	—	—	—	—	—
Dried distillers grain solubles, %	14.87	15.07	14.89	14.89	14.12	14.12	15.33	15.33
Suspended supplement^3^, %	4.93	5.00	4.93	4.93	5.26	5.26	5.31	5.31
Analyzed composition								
Dry matter, %	54.56	54.33	54.58	54.58	63.25	63.25	77.99	77.99
Crude protein, %	13.52	13.54	13.52	13.52	13.54	13.54	14.31	14.31
Neutral detergent fiber, %	23.50	28.22	23.50	23.50	19.41	19.41	17.78	17.78
Acid detergent fiber, %	12.31	15.39	12.31	12.31	9.64	9.64	8.79	8.79
Ether extract, %	4.52	4.35	4.52	4.52	4.52	4.52	4.71	4.71
Ash, %	2.85	3.46	2.85	2.85	2.49	2.49	2.79	2.79
Net energy of maintenance, Mcal/kg	1.92	1.82	1.92	1.92	1.98	1.98	2.01	2.01
Net energy of gain, Mcal/kg	1.29	1.21	1.29	1.29	1.35	1.35	1.37	1.37

^1^SD-SD = heifers that originated from SD and were finished in a feedlot in SD; TX-SD = heifers that originated from Texas and were finished in a feedlot in SD.

^2^Ground corn + MGA 200 premix replaced a portion of dry-rolled corn (0.23 kg/hd daily premix) to include MGA at 0.50 mg/heifer daily.

^3^Suspended supplement (all values except DM on a DM basis): 69.04% DM, 41.86% Crude Protein, 38.38% NPN, 0.95 Mcal/kg NEm, 0.66 Mcal/kg NEg, 23.00% TDN, 0.91% Crude Fat, 0.43% Crude Fiber, 10.89% Ca, 0.32% P, 7.00% K, 0.22% Mg, 6.03% NaCl, 3.07% Na, 0.33% S, 4.23 ppm Co, 199.88 ppm Cu, 11.99 ppm I, 15.07 mg/kg EDDI, 83.16 ppm Fe, 304.81 ppm Mn, 2.90 ppm Se, 664.59 ppm Zn, 44,064.55 IU/kg Vit A, 376.52 IU/lg Vit E, and 638.63 g/mg monensin sodium (Rumensin 90; Elanco Animal Health, Greenfield, IN).

**Table 2.** Formulated and analyzed diet composition of heifers fed in the Texas for 78 d^1^

	Days 1 to 12		Days 13 to 17		Days 18 to 22		Days 23 to 78	
Item	TX-TX	SD-TX	TX-TX	SD-TX	TX-TX	SD-TX	TX-TX	SD-TX
Steam-flaked corn^2^, %	61.99	20.35	61.99	34.66	61.99	49.62	61.99	61.99
Sweet Bran, %	27.50	55.91	27.50	45.23	27.50	34.96	27.5	27.50
Alfalfa, %	6.00	19.67	6.00	15.52	6.00	10.19	6.00	6.00
Limestone, %	2.51	1.98	2.51	2.59	2.51	2.53	2.51	2.51
Dry supplement^3^, %	1.50	2.09	1.50	2.00	1.50	2.04	1.50	1.50
Urea, %	0.50	---	0.50	---	0.50	0.66	0.50	0.50
Analyzed composition								
Dry matter, %	76.90	80.10	76.90	71.40	76.90	76.60	76.90	76.90
Crude protein, %	13.30	17.30	13.30	16.60	13.30	14.80	13.30	13.30
Neutral detergent fiber, %	19.30	29.90	19.30	30.80	19.30	23.50	19.30	19.30
Acid detergent fiber, %	8.20	16.50	8.20	17.10	8.20	12.90	8.20	8.20
Ether extract, %	3.00	2.50	3.00	2.40	3.00	2.60	3.00	3.00
Ash, %	4.80	11.60	4.80	10.70	4.80	8.70	4.80	4.80
Net energy of maintenance, Mcal/kg	2.25	1.90	2.25	1.90	2.25	2.12	2.25	2.25
Net energy of gain, Mcal/kg	1.54	1.21	1.54	1.21	1.54	1.43	1.54	1.54

^1^SD-TX = heifers that originated from SD and were finished in a feedlot in Texas; TX-TX = heifers that originated from Texas and were finished in Texas.

^2^Ground corn + MGA 200 premix replaced a portion of steam-flaked corn (0.23 kg/hd/d premix) to include MGA at 0.50 mg/heifer daily.

^3^Vitamins and minerals met or exceeded NASEM (2016) requirements for finishing beef heifers and included monensin sodium (Rumensin 90; Elanco Animal Health, Greenfield, IN) at 33.1 g/mg. Supplement supplied 5.99% potassium chloride, 44.40% crude protein, 3.82% sodium, 8.34 mg/kg cobalt carbonate, 395.00 mg/kg copper sulfate, 408.00 mg/kg iron sulfate, 764 mg/kg manganous oxide, 2.92 mg/kg selenium, and 2,490.00 mg/kg zinc sulfate on a DM basis. Actual diet formulation based on weekly DM determinations.

### Health measures

Clinical attitude scores (**CAS**) were recorded on days −1, 0, 1, 2, and 3 to monitor herd health for symptoms of bovine respiratory disease (**BRD**). These scores were based on a 0 to 3 scale: 0 = normal, 1 = mild BRD, 2 = moderate BRD, and 3 = severe BRD (Love et al., 2014).

Vaginal temperature probes (Burdick et al., 2012) were inserted into heifers to record continuous temperature at 5-min intervals to monitor stress response in heifers that were transported or not transported, without adding additional stress to the heifers from handling or presence of humans. Probes were inserted into all heifers that were transported (50 from SD-TX and 46 from TX-SD) and a portion of the heifers that remained in their respective state of origin for finishing (34 from SD-SD and 30 from TX-TX). Data loggers that had fallen out or did not record data were not included in the data set.

### Weather measurement and temperature-humidity index estimation

Climatic variables (ambient temperature, relative humidity, and wind speed) were obtained every 30 minutes from a weather station located near the RNC and Burnett Center prior to and throughout the experimental period (June 2022 through October 2022). The temperature-humidity index (**THI**) was calculated using the formula: THI = 0.81 × ambient temperature + [relative humidity × (ambient temperature—14.40)] + 46.60 (Hahn, 1999). The Livestock Weather Safety Index (LCI, 1970) classifications for heat stress include: ≤ 74 = normal; 75 ≤ THI ≥ 78 = alert; 79 ≤ THI ≥ 83 = danger; and ≥ 84 = emergency. Therefore, a baseline average THI value of 75 was used to determine when heifers were experiencing high ambient temperature loads.

### Growth performance

All heifers were weighed on days −1, 3, 15, 28, 56, 78 (TX finished heifers only), and 90 (SD finished heifers only). Heifers in SD-TX and TX-SD groups were also weighed on day 1 following transport to their respective finishing location to determine transportation shrink. Samples for microbial analysis were collected during these same timepoints and the results are discussed by Dornbach et al., (2023). Heifers finished in SD were on feed for an additional 12 d because of staging availability at the commercial abattoir. Incremental growth performance measures were calculated between BW collection days. Cumulative growth performance was based on BW from day −1 (with a 4% pencil shrink applied to account for digestive tract fill) and the final BW (shrunk 4%). Average daily gain (**ADG**) was calculated as the difference between BW and initial shrunk BW, divided by the days on feed for the respective period; gain efficiency was calculated from ADG divided by dry matter intake (**DMI**). DMI as a percentage of BW was calculated from the average DMI divided by BW.

### Carcass characteristics

On day 79 TX-TX and SD-TX, and day 90 for SD-SD and TX-SD, heifers were shipped from their finishing location in the afternoon to a commercial abattoir. Heifers were harvested the following morning and carcass data was collected after chilling approximately 24 h. Hot carcass weight (**HCW**) was recorded prior to entry to the chilling cooler. Camera data was collected from the harvest facility for the ribeye area, 12th rib fat, and marbling scores. USDA Yield Grade (**YG**) and USDA Quality Grade (**QG**) were evaluated according to the United States Standards for Grades of Carcass Beef (USDA, 2017). The dressing percentage was calculated as HCW divided by the final shrunk BW. Estimated empty body fat (**EBF**) percentage and final BW at 28% EBF (AFBW) were calculated from carcass traits (Guiroy et al., 2002) and the retail yield (**RY**) was calculated from the equation to determine the proportion of closely trimmed boneless retail cuts from the round, loin, rib, and chuck of a carcass (Murphey et al., 1960). Liver abscess severity and prevalence were recorded according to the Elanco Liver Scoring System: normal (no abscesses), A- (1 or 2 small abscesses or abscess scars), A (2 to 4 well-organized abscesses less than 2.54 cm. diameter), or A + (1 or more large active abscesses greater than 2.54 cm. diameter with inflammation of surrounding tissue).

### Statistical analysis

The experimental design was a completely randomized design with a 2 × 2 factorial arrangement of state of origin and state of finishing. For CAS and body temperature data, the individual heifer was designated as the experimental unit. Pen was considered the experimental unit when evaluating growth performance and carcass characteristics. The GLIMMIX procedure of SAS 9.4 (SAS Inst., Cary, NC) was used to evaluate growth performance, carcass traits, and multinomial data, with fixed effects of source of origin and source of finishing and their interaction. Pen location within each finishing facility was considered as a random effect. Initial BW was included as a covariate in the model. The MIXED procedure of SAS was used for temperature data analysis with time included as a repeated measure. The Kenward Roger adjustment was used to correct the degrees of freedom for unequal experimental units per treatment. Least squares means were separated using the Tukey option in the LSMEANS statement of SAS. An α ≤ 0.05 was considered significant and tendencies were discussed at 0.05 < α ≤ 0.10.

## Results

### Health measures and temperature data

Clinical attitude score data can be viewed in Table 3. On days −1 and 2, no state of origin × state of finishing interactions (*P* > 0.83) were observed in the distribution of CAS. On day 0, there was a shift in the distribution (*P* = 0.05) of CAS with more scores of 1 and 2 in the TX-TX heifers than all other treatments, which had a score of 0. On day 1, TX-TX and SD-TX heifers had a greater shift in the distribution (*P* = 0.02) towards scores of 1 and 2 than TX-SD and SD-SD heifers, with SD-SD heifers presenting a CAS of 0. On day 3, all treatments had a CAS of 0 and 1, but TX-TX heifers had a greater proportion (*P* = 0.03) of heifers with a CAS of 1 compared to all other treatments.

**Table 3.** Effect of source of origin (SD vs. TX) and finishing location (SD vs. TX) on heifer clinical attitude scores (CAS^1^) prior to and following transit^2^

Day	CAS	SD-SD	SD-TX	TX-SD	TX-TX	Org × Fin^3^
−1	0	100.0	100.0	82.6	80.4	0.83
	1	0.0	0.0	17.4	15.2	
	2	0.0	0.0	0.0	2.2	
	3	0.0	0.0	0.0	2.2	
Day 0	0	100.0	100.0	100.0	76.1	0.05
	1	0.0	0.0	0.0	19.6	
	2	0.0	0.0	0.0	4.3	
Day 1	0	100.0	92.0	97.8	80.4	0.02
	1	0.0	6.0	2.2	15.2	
	2	0.0	2.0	0.0	4.4	
Day 2	0	100.0	90.0	95.6	89.1	0.52
	1	0.0	6.0	2.2	8.7	
	2	0.0	4.0	2.2	2.2	
Day 3	0	97.9	96.0	93.5	92.6	0.03
	1	2.1	4.0	6.5	17.4	

^1^CAS recorded on days −1, 0, 1, 2, and 3 to observe heifers for symptoms of illness on a 0 to 3 scale: 0 = normal, 1 = mild BRD, 2 = moderate bovine respiratory disease, and 3 = severe BRD.

^2^SD-SD = heifers that originated from SD and were finished in a feedlot in South Dakota SD-TX = heifers that originated from SD and were finished in a feedlot in Texas; TX-SD = heifers that originated from Texas and were finished in a feedlot in SD; TX-TX = heifers that originated from Texas and were finished in Texas.

^3^Source of origin by finishing location interaction.

**Figure 1.**
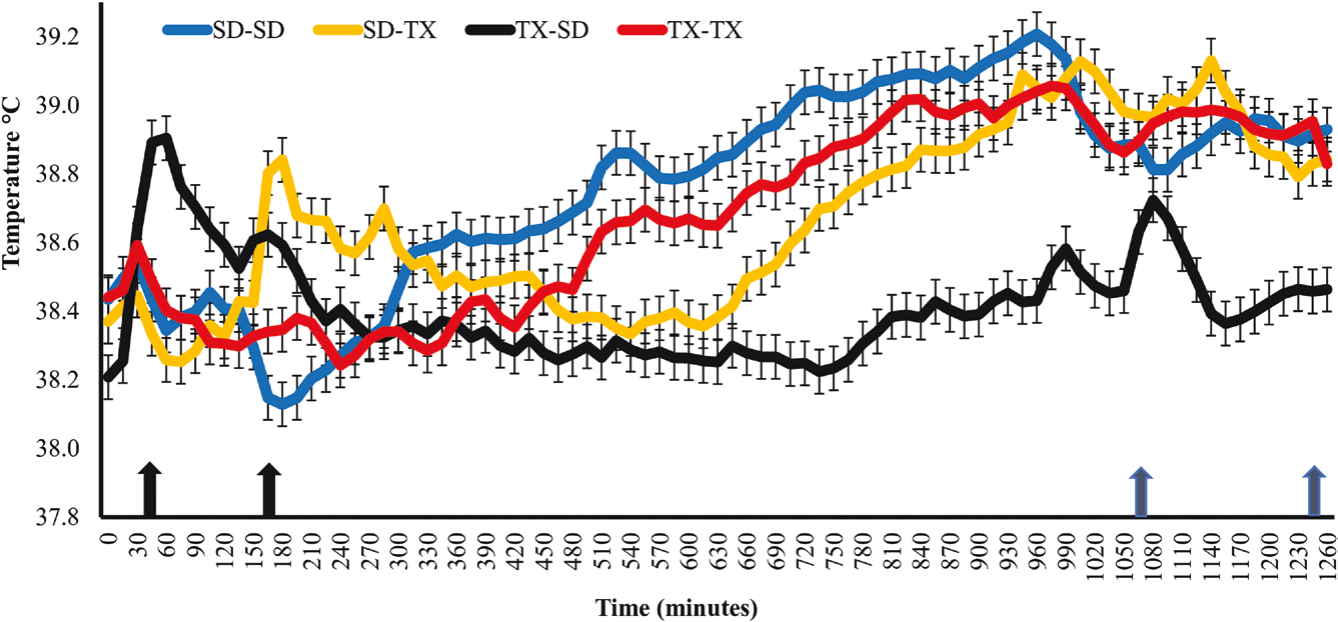
Vaginal temperatures during transit of heifers that remained in their state of origin (SD-SD or TX-TX) or were transported to another state for finishing (SD-TX or TX-SD). SD-SD = heifers that originated from SD and were finished in a feedlot in South Dakota SD-TX = heifers that originated from SD and were finished in a feedlot in Texas; TX-SD = heifers that originated from Texas and were finished in a feedlot in SD; TX-TX = heifers that originated from Texas and were finished in Texas. Timepoint 0 was 4:00 a.m. on 7/19/22. Timepoint 45 was when TX-SD heifers were shipped (4:45 a.m.) and timepoint 165 (6:45 a.m.) was when SD-TX heifers were shipped (left two black arrows). Timepoint 1065 was when TX-SD heifers arrived in SD (9:45 pm) and timepoint 1,245 (12:45 a.m.) was when SD-TX heifers arrived in TX (right two blue arrows). Treatment × Time (*P* = 0.01), Treatment (*P* = 0.01), and Time (*P* = 0.01).

There was a treatment × time interaction (*P* = 0.01) for heifer vaginal temperatures during transit (Figure 1). Transit temperature includes temperatures recorded approximately one hour prior to shipment (timepoint 0) until both SD-TX and TX-SD heifers arrived at their respective finishing location (timepoint 1260). Vaginal temperature increased (*P* ≤ 0.01) during the time of handling and loading for the TX-SD (timepoint 45, 0445 h) and SD-TX (timepoint 165, 0645 h) heifers. In general, the vaginal temperature of transported heifers remained lower (*P* ≤ 0.01) than heifers that were not transported. Vaginal temperature in SD-TX heifers increased (*P* ≤ 0.05) after timepoint 645. Vaginal temperatures of heifers remaining in their state of origin began to rise (*P* ≤ 0.05) at approximately timepoint 495 (1225 h) and reached a peak at approximately timepoint 960 (2000 h; SD-SD) and timepoint 975 (2015 h; TX-TX). Temperatures of heifers that were transported increased (*P* ≤ 0.05) again following unloading and handling for TX-SD (timepoint 1065; 2145 h) and SD-TX (timepoint 1245; 0045 h) heifers.

**Figure 2.**
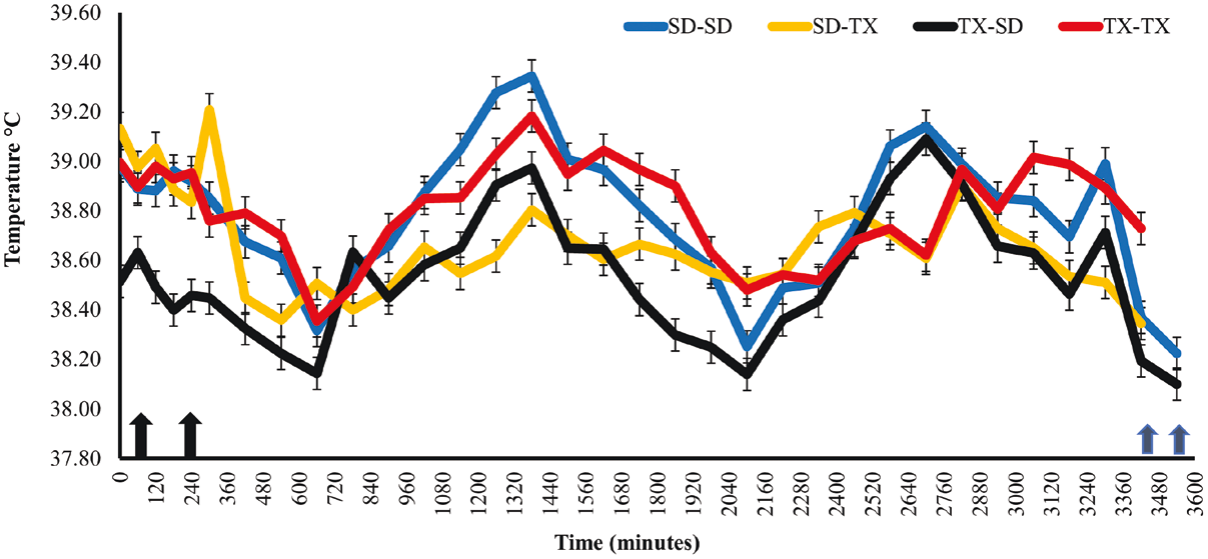
Vaginal temperatures post-transit of heifers that remained in their state of origin (SD-SD or TX-TX) or were transported to another state for finishing (SD-TX or TX-SD). SD-SD = heifers that originated from SD and were finished in a feedlot in South Dakota SD-TX = heifers that originated from SD and were finished in a feedlot in Texas; TX-SD = heifers that originated from Texas and were finished in a feedlot in SD; TX-TX = heifers that originated from Texas and were finished in Texas. Timepoint 0 was 8:45 p.m. on 7/19/22. Timepoint 60 was when TX-SD heifers arrived (9:45 p.m.) and were unloaded in SD and timepoint 240 (14:45 a.m. on 7/20/22) was when SD-TX heifers arrived and were unloaded in TX (left two black arrows). Timepoint 3,420 is when heifers in TX (6:45 a.m. on 7/22/22) were worked to remove temperature probes and timepoint 3,525 (7:45 a.m. on 7/22/22) is when heifers in SD were worked to remove temperature probes (right two blue arrows). Treatment × Time (*P* = 0.01), Treatment (*P* = 0.01), and Time (*P* = 0.01).

There was a treatment × time interaction (*P* ≤ 0.01) for heifer vaginal temperature data post-transit (Figure 2). Post-transit temperature includes temperatures recorded approximately 1 hour before SD-TX and TX-SD heifer feedlot arrival (timepoint 0) until all treatments were handled to remove the temperature probes (timepoint 3600). After arrival at each finishing location, temperatures of heifers that were transported (SD-TX and TX-SD) were lower (*P* ≤ 0.01) on day 1 compared to heifers that were not transported and differed (*P* ≤ 0.01) compared to their counterparts being finished in the same state. By day 2, heifers finished in the same state did not differ (*P* > 0.55) in vaginal temperatures, and heifers finished in TX had lower (*P* ≤ 0.02) vaginal temperatures compared to heifers in SD.

**Figure 3.**
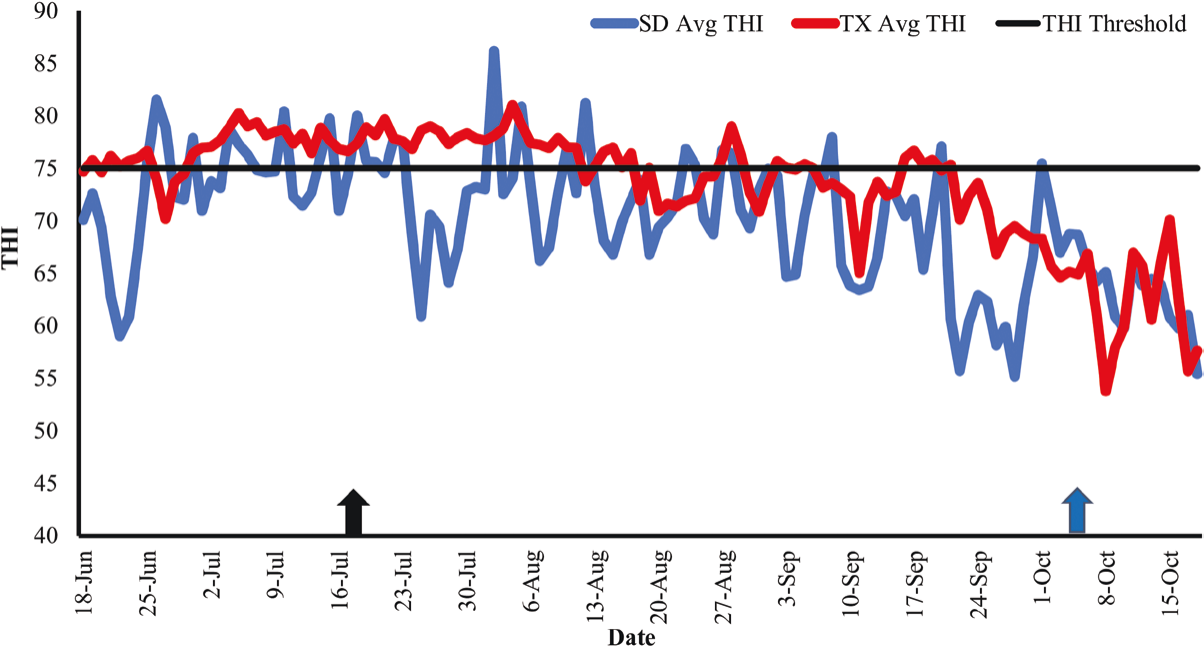
Average temperature-humidity index (THI) during the feeding period at the Ruminant Nutrition Center in Brookings, SD (blue line; SD Avg THI) and at the Burnett Center in Lubbock, TX (red line; TX Avg THI). THI was calculated as THI = 0.81 × ambient temperature, °C + [relative humidity × (ambient temperature, °C—14.40)] + 46.40 according to Hahn, 1999. The black arrow on the left indicates study initiation. The blue arrow on the right indicates when heifers at the Burnett Center in Lubbock, TX were shipped to be harvested. Heifers in TX were shipped 2 wk earlier than SD finished heifers because of packing plant availability. Values above the THI baseline of 75 were considered heat stress. Heifers in TX were exposed to elevated THI at 54% of the feeding period and heifers in SD were exposed to elevated THI at 18% of the feeding period.

The average temperature-humidity index (THI) is presented in Figure 3. A THI baseline of 75 was used to determine if heifers were experiencing high ambient temperatures. The THI in TX remained above the established threshold for heat stress approximately 1 month after study initiation without deterring below that threshold. The THI in SD varied throughout the study. Overall, heifers finished in TX were exposed to a THI above the established heat stress threshold level for 54% of the finishing period, and heifers in SD were exposed to a THI above the established threshold level for 18% of the feeding period.

### Growth performance

Growth performance responses are presented in Table 4. There was no state of origin × state of finishing interactions (*P* > 0.34) for day −1 BW and transit shrink. There was a state of origin × state of finishing interaction for day 3 BW and days 4 to 15 BW, ADG, DMI, gain:feed, and DMI as a percentage of BW. Heifer BW on days 3 and 15 were different (*P* ≤ 0.01) among treatments (SD-SD > SD-TX > TX-TX > TX-SD). The SD-SD heifers gained less (*P* ≤ 0.01) than TX-SD heifers, which also gained less (*P* ≤ 0.01) than heifers finished in TX, which did not differ (*P* = 0.60). The SD-SD heifers consumed less feed (*P* ≤ 0.01) than TX-TX heifers but consumed more feed (*P* ≤ 0.01) than transported heifers (TX-SD and SD-TX), which did not differ (*P* = 0.92). Heifers sourced in TX were less efficient (*P* ≤ 0.01) compared to SD-TX heifers but had increased gain:feed (*P* ≤ 0.01) compared to SD-SD heifers. Heifers finished in SD consumed less feed as a percentage BW (*P* ≤ 0.01) than TX-TX heifers but consumed more (*P* ≤ 0.01) than SD-TX heifers.

**Table 4.** Effect of source of origin (SD vs. TX) and finishing location (SD vs. TX) on cumulative growth performance responses^1^

	Treatment^2^					*P*-value		
Item	SD-SD	TX-SD	SD-TX	TX-TX	SEM	Origin	Finish	Org × Fin
Pens, *n*	6	6	5	5	—	—	—	—
Heifers, *n*	48	46	50	46	—	—	—	—
Initial BW (day −14), kg	48	425	483	425	1.2	—	—	—
Day −1 BW, kg	480	438	478	430	4.1	0.01	0.08	0.34
Day 1 BW, kg	—	411^a^	449^b^	—	3.6	—	—	0.01
Transit shrink, %	—	-6.28	-6.51	—	0.419	—	—	0.60
Day 3 BW, kg	480^d^	414^a^	454^c^	437^b^	3.6	0.01	0.57	0.01
Day 4 to 15								
BW, kg	495^c^	437^a^	488^c^	468^b^	3.8	0.01	0.01	0.01
Average daily gain (ADG), kg/d	1.34^a^	2.06^b^	2.95^c^	2.70^c^	0.200	0.10	0.01	0.01
Dry matter intake (DMI), kg	9.59^b^	8.48^a^	9.78^b^	10.65^c^	0.173	0.36	0.01	0.01
G:F	0.14^a^	0.24^b^	0.34^c^	0.25^b^	0.021	0.04	0.01	0.01
DMI % of BW	1.94^b^	1.94^b^	2.00^a^	2.28^c^	0.030	0.01	0.01	0.01
*Day 16 to 28*								
BW, kg	520^d^	458^a^	511^c^	498^b^	4.1	0.01	0.01	0.01
ADG, kg/d	1.87^a,b^	1.60^a^	1.79^a,b^	2.31^b^	0.235	0.44	0.07	0.02
DMI, kg	10.14^b^	9.69^a^	9.17^a^	11.00^c^	0.191	0.01	0.20	0.01
G:F	0.19	0.17	0.19	0.21	0.023	0.91	0.10	0.27
DMI % of BW	1.95^b^	2.12^c^	1.92^a^	2.35^c^	0.038	0.01	0.19	0.01
Day 29 to 56								
BW, kg	564^c^	519^a^	547^b^	545^b^	5.7	0.01	0.33	0.01
ADG, kg/d	1.56	2.19	1.27	1.66	0.147	0.01	0.01	0.26
DMI, kg	11.34^b^	12.03^b^	10.04^a^	11.89^b^	0.362	0.01	0.01	0.02
G:F	0.14^a^	0.18^b^	0.13^a^	0.14^a^	0.011	0.01	0.01	0.04
DMI % of BW	2.01	2.32	1.84	2.18	0.051	0.01	0.01	0.36
*Day 57 to finish* ^2^								
BW, kg	622^b^	587^a^	577^a^	585^a^	6.0	0.01	0.33	0.01
ADG, kg/d	1.72	1.98	1.39	1.84	0.081	0.01	0.01	0.26
DMI, kg	11.97	13.16	10.12	11.94	0.402	0.01	0.01	0.27
G:F	0.14	0.15	0.14	0.15	0.010	0.10	0.91	0.44
DMI % of BW	1.92	2.24	1.75	2.04	0.055	0.01	0.01	0.64
*Cumulative*								
ADG, kg/d	1.63	1.99	1.65	1.97	0.054	0.01	0.96	0.71
DMI, kg	10.76^b^	10.84^b^	9.78^a^	11.37^b^	0.242	0.01	0.20	0.01
G:F	0.15^a^	0.18^b^	0.17^b^	0.17^b^	0.004	0.01	0.25	0.01
DMI % of BW	1.73^b^	1.85^c^	1.69^a^	1.94^d^	0.032	0.01	0.17	0.01

^1^A 4% pencil shrink was applied to all BW measures to account for gastrointestinal tract fill.

^2^SD-SD = heifers that originated from SD and were finished in a feedlot in South Dakota SD-TX = heifers that originated from SD and were finished in a feedlot in Texas; TX-SD = heifers that originated from Texas and were finished in a feedlot in SD; TX-TX = heifers that originated from Texas and were finished in Texas.

^3^Final body weights measured either at day 79 for SD-TX and TX-TX and at a day 90 for TX-SD and SD-SD because of packing plant availability.

^a,b,c,d^Means within a row without a common superscript differ (*P *< 0.05).

There was a state of origin × state of finishing interaction for days 16 to 28 BW, ADG, DMI, and DMI as a percentage of BW. Heifer BW on day 28 differed (*P* ≤ 0.01) among treatments (SD-SD > SD-TX > TX-TX > TX-SD). Specifically, TX-SD heifers gained less (*P* < 0.03) than TX-TX heifers but were not different (*P* ≥ 0.59) to SD sourced heifers, which were not different than TX-TX (*P* ≥ 0.15). The SD-TX and TX-SD heifers did not differ in DMI (*P* > 0.28) but ate less (*P* ≤ 0.01) than SD-SD heifers, which also ate less (*P* ≤ 0.01) than TX-TX heifers. Heifers sourced in TX did not differ (*P* > 0.06) in DMI as a percentage of BW but were greater (*P* ≤ 0.01) than SD-SD heifers, which were also greater (*P* ≤ 0.01) than SD-TX heifers.

There was a state of origin × state of finishing interaction for days 29 to 56 BW, DMI, and gain efficiency. The TX-SD heifers were lighter (*P* ≤ 0.01) than heifers finished in TX, which did not differ (*P* > 0.05); heifers finished in TX were lighter (*P* ≤ 0.01) than SD-SD heifers. The SD-TX heifers consumed less (*P* ≤ 0.01) feed compared to all other treatments, which were not different (*P* > 0.05). The TX-SD heifers had greater gain:feed (*P* ≤ 0.01) than all other treatments, which did not differ (*P* > 0.05).

There was a state of origin × state of finishing interaction for final BW, cumulative DMI, cumulative gain:feed, and cumulative DMI as a percentage of BW. The SD-SD heifers had the heaviest (*P* < 0.01) final BW compared to all other treatments, which were not different (*P* ≥ 0.35). The SD-TX had decreased (*P* ≤ 0.01) cumulative DMI compared to SD-SD, TX-SD, and TX-TX heifers, which were not different (*P* > 0.09). The SD-SD heifers had decreased (*P* ≤ 0.01) cumulative gain efficiency than all other treatments, which were not different (*P* ≥ 0.11). Cumulative DMI as a percentage of BW was different (*P* = 0.01) among treatments (TX-TX > TX-SD > SD-SD > SD-TX). There was no state of origin × state of finishing interaction for cumulative ADG (*P* = 0.71), however, TX-sourced heifers had greater (*P* = 0.01) ADG than SD-sourced heifers.

### Carcass characteristics

Carcass measures are presented in Table 5. There was a state of origin × state of finishing interaction for HCW, 12th ribfat, marbling score, calculated YG, RY, EBF, AFBW, USDA YG, and USDA QG. The SD-SD heifers had heavier (*P* ≤ 0.01) HCW than heifers in all other treatments, which were not different (*P* ≥ 0.74). The SD-SD heifers had greater (*P* ≤ 0.01) 12th rib fat than heifers in all other treatments, which were not different (*P* ≥ 0.87). The SD-SD heifers had greater (*P* ≤ 0.01) marbling scores than TX-SD and SD-TX heifers, which were not different (*P* = 0.13), and TX-TX heifers, which were not different from SD-TX heifers (*P* = 0.34). Calculated YG and EBF were not different (*P* > 0.93) between TX-TX and SD-TX heifers but were less (*P* ≤ 0.02) than TX-SD heifers, which were also less (*P* ≤ 0.01) than SD-SD heifers. The SD-SD heifers had heavier (*P* ≤ 0.01) AFBW than heifers in all other treatments, which were not different (*P* ≥ 0.15). RY was not different (*P* = 0.98) between TX-TX and SD-TX heifers but was greater (*P* ≤ 0.01) than TX-SD heifers, which was also greater (*P* ≤ 0.01) than SD-SD heifers.

**Table 5.** Effect of source of origin (SD vs. TX) and finishing location (SD vs. TX) on heifer carcass trait responses^1^

	Treatment^2^					*P*-value		
Item	SD-SD	TX-SD	SD-TX	TX-TX	SEM	Origin	Finish	Org × Fin
Hot carcass weight, kg	404^b^	367^a^	369^a^	364^a^	4.8	0.01	0.01	0.01
Dressing percent^3^, %	64.9	62.4	63.9	62.2	0.44	0.01	0.07	0.23
Ribeye area, cm sq	92.84	90.13	99.23	98.52	1.903	0.20	0.01	0.45
12th Rib fat, cm	1.78^b^	1.35^a^	1.30^a^	1.30^a^	0.069	0.01	0.01	0.01
Marbling^4^	621^c^	458^b^	417^a,b^	385^a^	18.3	0.01	0.01	0.01
Calculated yield grade^5^	3.53^c^	2.93^b^	2.40^a^	2.36^a^	0.123	0.01	0.01	0.01
Retail yield^6^, %	48.50^a^	49.94^b^	51.16^c^	51.26^c^	0.300	0.01	0.01	0.01
Empty body fat^7^, %	33.59^c^	29.68^b^	28.55^a^	28.27^a^	0.460	0.01	0.01	0.01
Adjusted final body weight^7^, kg	572^b^	546^a^	557^a^	552^a^	8.1	0.01	0.27	0.01
*Yield grade distribution, %*								
1	0.0^a^	6.5^a^	28.0^b^	23.9^b^		0.03	0.01	0.01
2	29.8^a^	60.9^b^	58.0^b^	56.5^b^				
3	55.3^c^	30.4^b^	14.0^a^	19.6^a,b^				
4	14.9^b^	2.2^a^	0.0^a^	0.0^a^				
*Quality grade distribution, %*								
Select	2.1^a^	23.9^b^	36.0^b^	63.0^c^		0.61	0.01	0.02
Low choice	19.2^a^	47.8^b,c^	58.0^c^	37.0^b^				
Premium choice	46.8^c^	26.1^b^	6.0^a^	0.0^a^				
Prime	31.9^b^	2.2^a^	0.0^a^	0.0^a^				
*Liver abscess severity and prevalence* ^8^ *, %*								
Normal	91.5	84.8	88.0	78.3		0.10	0.33	0.94
A−	6.4	8.7	8.0	10.9				
A+	2.1	6.5	4.0	10.8				

^1^Heifers finished in SD were on feed for 90 d and heifers finished in TX were on feed for 78 d.

^2^SD-SD = heifers that originated from SD and were finished in a feedlot in South Dakota SD-TX = heifers that originated from SD and were finished in a feedlot in Texas; TX-SD = heifers that originated from Texas and were finished in a feedlot in SD; TX-TX = heifers that originated from Texas and were finished in Texas.

^3^DP = (HCW/final BW shrunk 4%) × 100.

^4^300 = slight^00^ 400 = small^00^ 500 = Modest^00^ 600 = Moderate^00^.

^5^According to the regression equation described by USDA (2017).

^6^As a percentage of HCW according to Murphey et al. (1960).

^7^Calculated according the equations described by Guiroy et al. (2002).

^8^According to the Elanco Liver Scoring System: Normal (no abscesses), A− (1 or 2 small abscesses or abscess scars), A (2 to 4 well organized abscesses less than 1 in. diameter), or A + (1 or more large active abscesses greater than 1 in. diameter with inflammation of surrounding tissue).

^a,b,c^Indicate differences between treatments.

The TX-TX and SD-TX heifers had a shift (*P* ≤ 0.01) in the distribution of USDA YG towards a greater proportion of YG 1 and 2 carcasses than TX-SD and SD-SD heifers; SD-SD heifers had a shift (*P* ≤ 0.01) in the YG distribution with more YG 4 carcasses. The TX-TX and SD-TX heifers had a shift (*P *≤ 0.01) in the distribution of USDA QG with more USDA Select carcasses compared to SD-SD and TX-SD heifers, which had a greater (*P* ≤ 0.01) proportion of premium Choice and USDA Prime carcasses.

## Discussion

### Health measures and temperature data

The CAS were recorded to observe heifers for BRD symptoms (Love et al., 2014). One heifer from the TX-SD treatment was treated for symptoms of BRD and had a CAS of 3 on day 2. The environmental temperature in TX was a high of 41.7 °C (26.1 °C low) and in SD was a high of 36.1 °C (25.6 °C low) on the day of transit (day 0). The following day (day 1), the environmental temperature in TX reached a high of 40 °C (25.6 °C low), and in SD reached a high of 31.1 °C (18.3 °C low). Overall, the observed CAS did not indicate concern for BRD. Heifers finished in TX, regardless of state of origin, appeared to be more susceptible to environmental factors, which were likely the drivers for greater CAS. This is most likely caused by the increased temperatures heifers in this region experienced during this study week.

Vaginal temperatures have been reported to provide more accurate and less variable temperatures as opposed to rectal temperatures (Lees et al., 2018). In general, heifer vaginal temperatures appeared to follow the circadian rhythm of each day, with minimums experienced in the morning and late evening hours and maximums experienced in the afternoon hours (Kendall and Webster, 2009). Heifers that were transported had decreased vaginal temperatures compared to non-transported heifers. Vaginal temperatures of heifers that were not transported increased throughout the day, likely because of the heat of fermentation following the morning feeding (NASEM, 2016). Similar to Burdick et al., (2012), transported heifer temperatures in the present study were elevated during times of loading, unloading, and handling. Interestingly, the temperatures of the SD-TX heifers began to increase at timepoint 660 min; this corresponds with the approximate time that the semi-truck transporting heifers to TX reached western Kansas, and the environmental temperature was approximately 40 °C. The THI values during transit were 78 and 75 for TX and SD, respectively. Increased rectal temperatures have been observed at increasing THI values (Kim et al., 2023), which may help explain this increase in vaginal temperatures following the increase in environmental temperature throughout the day.

Increased THI values have been reported to have a positive correlation with incidences of heat stress in beef cattle (Mader, 2003). The severity of heat stress events may be more apparent in cattle that are exposed to increased thermal conditions during the day and are unable to experience nighttime cooling (Mader et al., 2006; Brown-Brandl, 2018). In the present study, heifers finished in TX had elevated THI values for 54% of the feeding period while heifers finished in SD only experienced elevated THI for 18% of the feeding period. The majority of the elevated THI values were observed during the first month of the study for heifers finished in TX. It was during this time that the average THI did not fall below the threshold level of 75, whereas in SD the average THI was below the threshold level the majority of the time. Although no heat stress measures in this study were recorded, it is possible that heifers finished in TX could have experienced greater heat stress than their counterparts in SD as evidenced by the amount of time spent in THI above 75. Zimbelman et al., (2009) reevaluated the THI value at which high-producing lactating dairy cows began experiencing symptoms of heat stress and determined that milk production was decreased at a THI value of 68 as opposed to 72. Perhaps high-producing beef cattle also experience this same effect, and THI threshold levels below 75 should be considered. The susceptibility to heat stress will likely increase as genetic selection for increased growth continues (Bernabucci et al., 2010).

### Growth Performance

The authors speculate that most of the differences in growth performance measures within the period were likely attributed to compensatory growth measures. Compensatory growth has been defined as a period of a faster or more efficient rate of growth following a period of a slower or less efficient rate of growth that could result from the nutritional or environmental stress of planned management strategies (NASEM, 2016). In the present study, both sets of heifers had experienced different planned nutritional management strategies with TX-sourced heifers grazing wheat pasture and SD-sourced heifers limit-fed a high-concentrate diet. Differences in backgrounding management are a limitation of this study. Nonetheless, backgrounding management does provide a realistic situation of cattle sourced from each respective region as only 30% of cattle backgrounded in a Northern Plains system are placed in a grazing-based system (Asem-Hiablie et al., 2016).

Pritchard (1996) described compensatory growth as influencing 2 production characteristics: increased DMI compared to cattle of the same BW and improved feed efficiency. The effects of this mechanism are especially apparent in the d 4 to 15 period where the TX-SD, SD-TX, and TX-TX heifers had increased gain:feed compared to SD-SD heifers. These differences were driven by decreased ADG with increased DMI for the SD-SD heifers. In the following period (d 16 to 28), there were no differences in gain:feed among treatments. The efficiencies observed from days 4 to 15 are partially because of the recovery of growth following transit for the SD-TX and TX-SD heifers as described by Pritchard and Preston (1992). The TX-TX heifers had increased gain:feed during this time, which may be explained by the heifers originating from TX provided a lower caloric diet during backgrounding compared to SD-sourced heifers (Drouillard et al., 1991). Before the start of this study, TX-sourced heifers likely had increased energy requirements for maintenance grazing wheat pasture compared to the SD-sourced heifers being limit-fed a high-concentrate ration, which led to enhanced utilization of the energy available for gain due to the increased DMI (Drouillard et al., 1991; Pritchard, 1996).

Since the heifers were transported at equal distances for similar durations, it is not surprising that transport shrink did not differ between TX-SD and SD-TX heifers. It was suggested by Self and Gay (1972) that cattle transported an average of 1,023 km have a 7% to 9% shrink and require approximately 10 d to recover BW lost during transportation. In the present study, heifers had 6.28% and 6.51% shrink for TX-SD and SD-TX heifers, respectively. The heifers in the present study were yearlings and did not appear to be highly stressed, as can be observed in newly weaned calves (Coffey et al., 2001), thus resulting in a lesser degree of shrink in heifers when transported 1,540 km. By d 15, heifers in both treatments recovered the transportation shrink, with TX-SD heifers weighing the same as on day −1 and SD-TX heifers weighing 10 kg more than on day −1. The difference in BW could explain the magnitude of increased ADG during the d 4 to 15 period for both of these treatments.

Cattle fed a steam-flaked corn-based diet have increased ADG and increased gain:feed than cattle fed a dry-rolled corn-based diet (Barajas and Zinn, 1998; Owens and Gardner, 2000; Corona et al., 2005). However, this phenomenon was not observed with growth performance in the present trial. Growth performance responses in the present trial did not appear to be dependent on the diet offered in each finishing region.

The growth recovered following transit has been termed recovered growth (Pritchard and Preston, 1992). It is evident that heifers did regain the growth lost during transit within 15 d, with SD-TX heifers not differing from the SD-SD heifers. However, the TX-SD heifers were lighter than the TX-TX heifers. At the start of the study, BW were similar within the source of origin but different between sources, so it is not surprising that there are differences in BW throughout the study between origin sources. When heifers were transported to greater ambient temperatures, this resulted in decreased cumulative DMI in SD-TX heifers. For the final BW at the end of the study, the SD-TX heifers weighed approximately 45 kg less than the SD-SD heifers, whereas the TX-SD heifers finished 2 kg heavier than the TX-TX heifers. The SD finished heifers had an additional 12 days on feed. Assuming heifers finished in TX gained similarly in the last period, the additional 12 days on feed would decrease the magnitude of BW differences between the SD-SD and SD-TX heifers (622 vs. 594 kg, respectively), but increase the magnitude between TX-TX and TX-SD heifers (607 vs. 587 kg, respectively). Unfortunately, because of harvest availability at the packing plants in each region during this time, it was not possible to harvest on the same days on feed, and the authors realize this is a limitation of this study.

### Carcass characteristics

There is limited research investigating the influence of transit to varying environments on carcass characteristics, as most research has been evaluated shortly following the transit period. The likely basis of variation in growth performance is further observed in carcass measures and is a function of the differences observed between the different types of backgrounding systems, breeds of cattle, and the additional 12 days on feed. In general, cattle coming from a lower plane of nutrition will finish leaner and at decreased percentages of EBF at equal days on feed (Hogg, 1991; Pritchard, 1996). These concepts were observed in the present study where the SD-sourced heifers finished with greater 12th rib fat thickness than the TX-sourced heifers.

Baumgard and Rhoads (2013) suggested that cattle can lose body mass during times of heat stress because of the increased need for glucose for the immune system and increased respiration loss (i.e., water) during these events. Cattle therefore have an increased maintenance requirement, because of increased respiration rate. Increased maintenance requirements coupled with decreased DMI results in less intake of energy available for gain. In the present study, SD-TX heifers had lighter and leaner carcasses than the SD-SD heifers. The lighter and leaner carcasses could be a result of the phenomenon discussed by Baumgard and Rhoads (2013). There is also increased lipolysis during heat stress events, as the immune system requires more glucose to maintain homeostatic conditions (Kvidera et al., 2017). A possible glucose-sparing event could have occurred in the heifers finished in TX, as these heifers had decreased marbling scores and QG compared to their counterparts finished in SD. Heifers finished in TX also had increased REA compared to heifers finished in SD. Perhaps the increase in REA may be partially attributed to the nutrient prioritization in the hierarchy of tissues (van Milgen and Noblet, 2003; Baumgard and Rhoads, 2013), but it is more likely attributed to differences in cattle breed type or backgrounding management.

The REA influenced the differences in YG and RY as greater proportions of lean tissue resulted in numerically decreased YG at the same level of 12th rib fat with similar carcass weight (USDA, 2017). The SD-SD heifers had greater 12th rib fat and increased QG which could contribute to greater EBF. Based on the results of the present study, it appears that heifers finished in greater ambient temperatures resulted in improved YGs, but also had decreased quality carcasses, with only 3% of the carcasses finished in TX grading premium choice or better. Moreover, heifers finished in decreased ambient temperatures had increased carcass quality but decreased cutability. As previous research has indicated that the ideal amount of 12^th^ rib fat needed to allow for optimal marbling is approximately 1.27 cm (Bruns et al., 2004; Maddock, 2013), it does not appear that the 12 fewer days on feed hindered the TX finished heifers from accumulating sufficient rib fat.

The TX-SD heifers had 26% of the carcasses grade premium choice or better with 24% USDA Select, compared to 0% premium choice or better with 63% USDA Select carcasses for the TX-TX heifers. These differences may be explained by the variations in the finishing diets between regions. It has been reported that cattle fed a steam-flaked corn diet compared to a dry-rolled corn-based diet had larger REA and greater rib fat, but decreased marbling scores and QG (Owens and Gardner, 2000). These findings are consistent with the results observed in the present study. The changes in carcass composition would reflect a shift in the site of digestion from the rumen (steam-flaked corn) to the small intestine (dry-rolled corn), resulting in increased subcutaneous fat deposition from less ruminal dietary starch for steam-flaked corn (Owens et al., 1997). The shift in site of the starch digestion mechanism could explain these differences in carcass measurements between cattle finished in different regions with differing corn processing methods.

Overall, treatments had less liver abscesses than the plant averages (overall mean prevalence 23% vs. plant average 30.8%) reported in the 2016 National Beef Quality Audit (Boykin et al., 2017). Decreases in liver abscesses in the present study coincide with previous research suggesting finishing heifers generally have lesser liver abscess prevalence than steers (Grimes, 2022). Heifers sourced from SD tended to have decreased liver abscess prevalence and severity than heifers sourced from TX. These differences are consistent with previous research indicating cattle in the Northern Plains region have a decreased incidence of liver abscess than those in the Southern Plains region (Grimes, 2022; Herrick et al., 2022).

## Implications

The type of backgrounding systems, breeds of cattle, and finishing diets fed in the current study represent applicable situations cattle producers and feeders experience between the two different finishing regions. The information presented in the current study attempted to consider these factors, but the authors realize that these factors could possibly contribute to the differences in growth performance and carcass characteristics observed in this study. Collectively, growth performance appeared to be influenced by backgrounding management experienced by the heifers (TX sourced heifers from wheat pasture vs. SD heifers sourced from grow yard with limit-fed high concentrate-based diet). Additionally, heifers transported from decreased ambient temperatures to greater ambient temperatures may have experienced an increased incidence of heat stress, which seemed to influence overall carcass merit. Carcass differences may also be partially caused by SD finished heifers being on feed for an additional 12 d. The type of processed grain (steam-flaked corn vs. dry-rolled corn) fed during the finishing phase may also affect growth performance and carcass quality. Therefore, further investigation is needed to conclude if transportation or ambient temperatures and the type of grain processing affect feedlot performance and overall carcass value in finishing cattle sourced and finished in different regions of the United States.

## Data Availability

Data can be made available upon reasonable request to ERD or ZKS.

## Abbreviations

ADG: average daily gain
AFBW: adjusted final body weight
BRD: bovine respiratory disease
BW: body weight
CAS: clinical attitude score
DMI: dry matter intake
EBF: empty body fat
EBW: empty body weight
THI: temperature-humidity index
RNC: ruminant nutrition center
RY: retail yield
